# Comparison of clinical outcomes between mesh-reinforced pancreatojejunostomy and pancreatogastrostomy following pancreaticoduodenectomy: a cohort study

**DOI:** 10.1186/s12957-018-1491-6

**Published:** 2018-09-17

**Authors:** Junhai Pan, Xiaolong Ge, Wei Zhou, Xin Zhong, Lihu Gu, Hepan Zhu, Xinlong Li, Weilin Qi, Xianfa Wang

**Affiliations:** Department of General Surgery, School of Medicine, Sir Run Run Shaw Hospital, Zhejiang University, 3 East Qingchun Road, Hangzhou, 310016 Zhejiang China

**Keywords:** Pancreatogastrostomy, Pancreatojejunostomy, Pancreaticoduodenectomy, Mesh, Pancreatic fistula

## Abstract

**Background:**

Postoperative complications, especially postoperative pancreatic fistulas, remain the major concern following pancreaticoduodenectomy (PD). Mesh-reinforced pancreatic anastomoses, including pancreatojejunostomy (PJ) and pancreatogastrostomy (PG), are a new effective technique in PD. This study was conducted to analyze the safety and efficacy of this new technique and to compare the results of mesh-reinforced PJ vs PG.

**Methods:**

A total of 110 patients who underwent PD between August 2005 and January 2016 were eligible in this study. Perioperative and postoperative data of patients with a mesh-reinforced technique were analyzed. Data were also grouped according to the procedure performed: mesh-reinforced PJ and mesh-reinforced PG.

**Results:**

Among patients undergoing PD with the mesh-reinforced technique, 42 had postoperative complications, and the comprehensive complication index (CCI) was 32.7 ± 2.5. Only 10% of patients had pancreatic fistula; three were grade A, six were grade B, and two were grade C. Biliary fistula occurred in only 8.2% of patients. Patients undergoing mesh-reinforced PG showed a significantly lower rate of CCI than did mesh-reinforced PJ patients (27.0 ± 2.1 vs 37.0 ± 3.9, *p* < 0.05). The mesh-reinforced PG was also favored over mesh-reinforced PJ because of significant differences in intra-abdominal fluid collection (5.9% vs 18.6%, *p* < 0.05) and delayed gastric emptying (3.9% vs 15.3%, *p* < 0.05).

**Conclusions:**

PD with the mesh-reinforced technique was a safe and effective method of decreasing postoperative pancreatic fistula. Compared with mesh-reinforced PJ, mesh-reinforced PG did not show significant differences in the rates of pancreatic fistula or biliary fistula. However, CCI, intra-abdominal fluid collection, and delayed gastric emptying were significantly reduced in patients with mesh-reinforced PG.

## Background

Pancreaticoduodenectomy (PD) is considered to be one of the most difficult and complex surgery since Whipple and colleagues published the first report of patients undergoing PD in 1935 [1]. In addition, PD has been regarded to be a standard surgical procedure in pancreatic cancer and other lesions which are located in periampullary region, while it is always with high postoperative complications rates [2–5]. Generally, postoperative pancreatic fistula (POPF), biliary fistula, intra-abdominal fluid collection, intra-abdominal hemorrhage, and delayed gastric emptying are common post-pancreaticoduodenectomy complications even there are advances in surgical techniques in recent years, and it leads to prolonged hospital stays and increased medical costs [6, 7]. Among all the postoperative complications, POPF is regarded as Achilles heel of PD. To prevent complications, various strategies have been tried including pharmacologic prophylactic approaches and surgical techniques [8, 9]. These include octreotide, occluding the main duct with rubber or fibrin glue, pancreatic duct stenting, suture ligation of the pancreatic duct, pancreaticoenterostomy with the jejunum or stomach, modification of suturing techniques, and others [10–12]. Although the rate of mortality after PD has decreased to less than 5%, the morbidity rate is still higher, ranging from 40 to 60% [13]. As the most harmful complications, the incidence of POPF has been reported, with highly variable rates depending on various definitions, ranging from 10 to 29% [14]. Postoperative complications at or around the anastomosis following PD were the most frequent and dangerous, occurring almost 25 times with five deaths [15]. Therefore, improvements in perioperative management and surgical technique remain crucial to decrease complications following PD.

For almost 10 years, we have used the new mesh-reinforced technique described by Peng and his colleagues to minimize postoperative complications after PD [16–18]. This safe and simple technique is characterized by a strip of polypropylene mesh, and it is wrapped around the pancreatic stump. Wang et al. [17] reported that no leakage occurred in 10 patients undergoing mesh-reinforced pancreaticojejunostomy (PJ) in PD. Only one patient developed grade A pancreatic leakage after mesh-reinforced pancreaticogastrostomy (PG) in a series of 13 initial cases [14, 18]. However, a large sample analysis is needed to evaluate whether the mesh-reinforced technique is safe and effective following PD. Some studies suggested that PG reduced biliary fistula and delayed gastric emptying and postoperative collections in patients following PD more so than did PJ [19, 20]. Therefore, whether mesh-reinforced PG is better than mesh-reinforced PJ in postoperative complications following PD remains unknown.

In this study, we evaluated the outcomes of the mesh-reinforced technique in 110 patients undergoing PD. Either PG or PJ was performed depending on the surgeon’s preference. Postoperative complications were assessed for both types of reconstruction.

## Methods

### Study patients

Consecutive patients undergoing pancreatoduodenectomy because of malignant or non-malignant disease were included and analyzed in a tertiary referral hospital between August 2005 and January 2016. Written informed consent which was associated with the potential surgical risks was signed by all the patients. The present research was approved by the ethics committee of our hospital.

### Surgical techniques

In our institution, we used the mesh-reinforced technique of pancreaticogastrostomy described by Professor Peng [16]. During the entire surgical procedure, non-absorbable (polypropylene mesh, large pore, Ethicon, New Jersey, USA) or absorbable (Cook, Limerick, Ireland) hernia grafts were used to reconstruct the pancreatic remnant. Pancreatojejunostomy and pancreatogastrostomy were the techniques used in reconstruction of pancreatic anastomosis. The mesh-reinforced technique in pancreaticogastrostomy was designed such that the pancreatic remnant wrapped in a mesh strip was embedded into the stomach and was bound to its posterior wall by a single layer of continuous sutures [18]. The mesh-reinforced technique in pancreaticojejunostomy was designed such that the sheath of the jejunum was bound to the pancreatic remnant which was wrapped by a strip of mesh. In our institution, this mesh-reinforced technique was the first choice for reconstruction following PD; it has been shown to be safe and effective for PG or PJ as described previously by Wang and Zhu [17, 18].

### Data collection

The data collection was performed retrospectively including baseline characteristics and laboratory data from the database in our hospital. In addition, baseline characteristics mainly included gender, age, body mass index (BMI), comorbidities, pancreatic gland texture, mesh materials, American Society of Anesthesiologists (ASA), and the surgical indication. Other data about surgery included operative time, intraoperative blood transfusion, intraoperative bleeding, and the size of pancreatic duct.

### Definition of outcomes

The primary outcome was pancreatic fistula after surgery according to the definition by International Study Group of Pancreatic Fistula (ISGPF). A clinical grading system of POPF was proposed as grades A, B, and C [14]. The comprehensive complication index (CCI) was created to evaluate postoperative complications, and the CCI was a score calculated based on the Clavien-Dindo system [21, 22]. Mild complications included grades I and II according to the Clavien-Dindo system, and the major complications included grades III to IV. Furthermore, the CCI system was reported to be more sensitive than the existing morbidity endpoints [21, 23]. The website of http://www.assessurgery.com was used to calculate the CCI in each patient [24]. Other outcomes included postoperative stay, biliary fistula, abdominal bleeding, intra-abdominal fluid collection, delayed gastric emptying, and reoperations [25].

### Statistical analysis

SPSS version 23.0 software was used in analyzing all the data. Continuous data were presented as mean ± SD, and categorical data were presented as number (%). The continuous variables were analyzed by Mann-Whitney *U* test or Student’s *t* test, and Pearson’s chi-square test or the Fisher exact test was used to analyze categorical variables. The statistically significance was defined as *P* value < 0.05.

## Results

### Patients

In total, 110 patients underwent pancreatic resections by the mesh-reinforced technique and were included in this retrospective observational analysis, of which 84.5% were confirmed to be soft parenchyma. Among 110 patients following PD, 25.5% had a dilated pancreatic duct with the diameter larger than 4 mm. Fifty-one patients underwent PG, and 59 patients underwent PJ with the new mesh-reinforced technique. In the PG group, there were 27 males and 24 females, and their mean age was 60.9 years. In the PJ group, the patients’ mean age was 57.1 years, and 38 patients were male. No statistically significant differences was found in the distributions of comorbidities, ASA, pancreatic gland texture, pancreatic duct size, age, and gender between the PG group and PJ group (Table 1). A total of 35 patients underwent PD due to ampullary carcinoma, 16 patients had duodenal cancer, 16 patients had distal biliary cancer, and 15 patients were diagnosed with ductal cancer. The remaining 28 patients had other conditions, primarily cystic tumors (9 patients), endocrine tumors (5 patients), and intraductal papillary mucinous tumors (5 patients). There was no significant difference between the PG group and the PJ group in terms of surgical indications (Table 2).

**Table 1:** Baseline demographics in patients undergoing pancreatectomy

Characteristic	All (*n* = 110)	PG group (*n* = 51)	PJ group (*n* = 59)	*P* value
Age*	58.9 ± 1.0	60.9 ± 1.3	57.1 ± 1.5	0.07
Men, *n* (%)	65 (59.1)	27 (52.9)	38 (64.4)	0.22
BMI*, kg/m^2^	23.0 ± 0.3	22.6 ± 0.6	23.3 ± 0.4	0.28
Comorbidities, *n* (%)				
Diabetes mellitus, *n* (%)	22 (20)	9 (17.6)	13 (22.0)	0.57
Hypertension, *n* (%)	27 (24.5)	13 (25.5)	14 (23.7)	0.83
Operation time*, min	368.0 ± 8.2	342.4 ± 11.7	390.0 ± 10.8	< 0.01
ASA ≥ 3, *n* (%)	19 (17.3)	9 (17.6)	10 (16.9)	0.92
Intraoperative blood transfusion, *n* (%)	41 (37.3)	14 (27.5)	27 (45.8)	0.048
Estimated blood loss*, mL	502.4 ± 48.3	392.2 ± 31.8	597.6 ± 84.2	0.03
Pancreatic gland texture, *n* (%)				0.55
Soft, *n* (%)	93 (84.5)	42 (82.4)	51 (86.4)	–
Hard, *n* (%)	17 (15.5)	9 (17.6)	8 (13.6)	–
Mesh materials, *n* (%)				< 0.01
Non-absorbable, *n* (%)	59 (53.6)	10 (19.6)	49 (83.1)	–
Absorbable, *n* (%)	51 (46.4)	41 (80.4)	10 (16.9)	–
Pancreatic duct size (> 4 mm), *n* (%)	28 (25.5)	14 (27.5)	14 (23.7)	0.66
The time of anastomosis*, min	27.4 ± 0.4	29.1 ± 0.7	27.9 ± 0.6	0.21

*PG* pancreatogastrostomy, *PJ* pancreatojejunostomy

*Value are expressed as the mean ± SE

**Table 2:** Surgical indications in 110 pancreaticoduodenectomies

	All (*n* = 110)	PG group (*n* = 51)	PJ group (*n* = 59)	*P* value
Ampullary carcinoma*	35 (31.8)	17 (33.3)	18 (30.5)	0.75
Distal biliary cancer*	16 (14.5)	7 (13.7)	9 (15.3)	0.82
Duodenal cancer*	16 (14.5)	7 (13.7)	9 (15.3)	0.82
Ductal cancer*	15 (13.6)	7 (13.7)	8 (13.6)	0.98
Cystic tumors*	9 (8.2)	4 (7.8)	5 (8.5)	0.90
Endocrine tumors*	5 (4.5)	3 (5.9)	2 (3.4)	0.53
Intraductal papillary mucinous tumor*	5 (4.5)	2 (3.9)	3 (5.1)	0.77
Other indications*	9 (8.2)	4 (7.8)	5 (8.5)	0.90

*PG* pancreatogastrostomy, *PJ* pancreatojejunostomy

*Values are expressed as *n* (%)

### Postoperative outcomes

After PD with the mesh-reinforced technique, 68 patients recovered uneventfully, and 42 patients suffered postoperative complications. Some patients were found to have more than two complications after surgery. According to the Clavien-Dindo system, there were 7 patients (6.4%) with mild complications (Clavien-Dindo grade I to II) and 35 patients (31.8%) with major complications (Clavien-Dindo grade III to IV). The mean CCI based on Clavien-Dindo classification was 32.7 ± 2.5. The mean operative time was 368.0 ± 8.2 min, with estimated blood loss of 502.4 ± 48.3 mL. The mean anastomosis time of PD with this mesh-reinforced technique was 27.4 ± 0.4 min. A total of 37.3% of all the patients received intraoperative blood transfusions, and the length of the postoperative stay was 23.2 ± 0.9 days.

### Postoperative pancreatic fistula

Various improvements in surgical technique were developed to avoid postoperative pancreatic fistula in studies regarding PD [13]. In our study, with the new mesh-reinforced technique, the rate of pancreatic fistula decreased to 10% in all the patients. According to the ISGPF definition [14], only three patients (2.7%) had grade A pancreatic fistula, six patients (5.5%) had grade B, and two patients (1.8%) had grade C (Table 3). In addition, only nine patients (8.2%) suffered biliary fistula, and eight patients (7.3%) had abdominal bleeding following PD. In addition, there were 14 patients (12.7%) with intra-abdominal fluid collections, and 11 patients (10.0%) had delayed gastric emptying. Finally, three patients (2.7%) underwent the reoperation due to serious complications associated with the pancreatic fistula (Table 3).

**Table 3:** Postoperative outcomes in 110 cases of pancreaticoduodenectomy

Outcome	All (*n* = 110)	PG group (*n* = 51)	PJ group (*n* = 59)	*P* value
Postoperative complication, n (%)	42 (38.2)	18 (35.3)	24 (40.7)	0.562
Mild complications, *n* (%)	7 (6.4)	3 (5.9)	4 (6.8)	0.848
Major complications, *n* (%)	35 (31.8)	15 (29.4)	20 (33.9)	0.614
CCI, mean ± SD	32.7 ± 2.5	27.0 ± 2.1	37.0 ± 3.9	0.045
Pancreatic fistula, *n* (%)	11 (10.0)	3 (5.9)	8 (13.6)	0.181
Pancreatic fistula grade				0.488
A, *n* (%)	3 (2.7)	1 (2.0)	2 (3.4)	–
B, *n* (%)	6 (5.5)	2 (3.9)	4 (6.8)	–
C, *n* (%)	2 (1.8)	0 (0)	2 (3.4)	–
Biliary fistula, *n* (%)	9 (8.2)	2 (3.9)	7 (11.9)	0.243
Postoperative stay, mean ± SD	23.2 ± 0.9	23.6 ± 1.6	22.8 ± 1.0	0.674
Abdominal bleeding, *n* (%)	8 (7.3)	4 (7.8)	4 (6.8)	0.830
Intra-abdominal fluid collection, *n* (%)	14 (12.7)	3 (5.9)	11 (18.6)	0.045
Delayed gastric empting, *n* (%)	11 (10.0)	2 (3.9)	9 (15.3)	0.048
Reoperations, *n* (%)	3 (2.7)	1 (2.0)	2 (3.4)	0.646

*PG* pancreatogastrostomy, *PJ* pancreatojejunostomy, *CCI* comprehensive complication index

### Comparison between PJ and PG with mesh-reinforced technique

At present, PJ and PG are both widely used following PD, and many studies have been published in order to compare the safety and efficacy of these two methods. Similarly, when the mesh-reinforced technique was performed during reconstruction in our study, the postoperative outcomes were compared between PJ and PG. The postoperative course showed that 18 (35.3%) patients had complications in the PG group and that 24 (40.7%) patients had complications in the PJ group. When compared to the PG group, there were statistically significant differences in the PJ group in terms of the comprehensive complication index (27.0 ± 2.1 vs 37.0 ± 3.9, *P* < 0.05). In the PG group, three patients (5.9%) had pancreatic fistulas, of which one patient (2.0%) had grade A and two patients (3.9%) had grade B. In the PJ group, eight patients (13.6%) had pancreatic fistulas, of which two patients (3.4%) were grade A, four patients (6.8%) were grade B and two patients (3.4%) were grade C. The PJ group was more likely to have biliary fistula than was the PG group, even though no significant difference was found (11.9% vs 3.9%). However, significant differences in favor of the PG group were investigated regarding intra-abdominal fluid collections (5.9% vs 18.6%, *P* < 0.05) and delayed gastric emptying (3.9% vs 15.3%, *P* < 0.05). The operative time was also significantly different between these two groups (PG, 342.4 ± 11.7 min vs PJ, 390.0 ± 10.8 min, *P* < 0.01), while the time of anastomosis was not significantly different between the PG and PJ groups. The PJ group also had significantly more estimated blood loss than the PG group. All the results are shown in Tables 1 and 3.

## Discussion

In this study, the safety and efficacy of mesh-reinforced pancreatojejunostomy and pancreatogastrostomy in patients following pancreatoduodenectomy were compared. We found that patients undergoing pancreatoduodenectomy with the mesh-reinforced technique suffered fewer postoperative complications, especially lower rates of pancreatic leakage. This retrospective study also indicated that PG with mesh-reinforced pancreatoduodenectomy resulted in fewer complications and improved the quality and safety of the pancreatic anastomosis more so than did the PJ with mesh-reinforced following PD.

The reported frequency of pancreatic fistula after pancreaticoduodenectomy varied from 10 to 30%, and pancreatic fistula was considered a serious event that even might be life-threatening in almost half of patients [15, 26, 27]. The reasons for pancreatic fistula were primarily its soft parenchyma, making it more likely to develop parenchymal lacerations from shear forces applied during tying of sutures [17]. In addition, the anastomotic technique, the diameter of pancreatic duct, the general condition of the patients and the various definitions of pancreatic fistula could also affect the incidence of postoperative pancreatic fistula [14, 28–30]. It was reported that soft pancreatic parenchyma and the diameter of main pancreatic duct less than 3 mm were risk factors for pancreatic leakage [31, 32]. To prevent these complications, various methods during the preoperative, intraoperative, and postoperative periods have been proposed, especially surgical techniques [33, 34]. For example, it is reported that the application of the novel embeddedness-like pancreaticojejunostomy anastomosis technique in PD was effective and could reduce the incidence of pancreatic fistula [35]. In our hospital, we also introduce a different method of mesh-reinforced technique in PD to reduce postoperative complications.

The rate of pancreatic fistula of 10% in our study was lower than that of other studies. The technique used in our procedure to reduce morbidity and mortality was modified according to the binding pancreaticojejunostomy described by Peng et al. [16]. There exist some advantages of this technique as previously described [36]. First, the safe anchor site was provided by the mesh for sutures, and this resulted in the ability to avoid leakage and bleeding after surgery for soft and fragile pancreatic tissue. Second, it was more convenient to wrap the pancreatic stump with the bowel loop by changing the shape of the pancreas with mesh reinforcement. In addition, the left edge of the mesh was sutured tightening to the posterior wall of the bowel loop, and it facilitated the pancreas sliding into the bowel loop. Fourth, the likelihood of pancreatic leakage and bleeding was diminished by the mesh compression of pancreatic tissue. Finally, the mesh stimulated the growth of fibroblasts and promoted the healing process between the pancreatic capsule and the bowel mucosa [36–38]. In our study, we proposed various mesh-reinforced surgical techniques to prevent complications, and there were lower rates of several surgical complications.

There are two methods for the restoration of pancreatic drainage into the gastrointestinal tract in PD, including PG and PJ [19, 39, 40]. Not all previous studies yielded similar results. Yeo et al. [41] reported that the rate of pancreatic fistula was almost the same for the PG (12.3%) and PJ (11.1%), suggesting that PG was not safer than PJ. However, Bassi et al. [19] found that biliary fistula, postoperative collections and delayed gastric emptying rates were significantly lower in patients with PG than in PJ. Schlitt et al. [20] also suggested that PG was significantly safer than PJ with respect to the incidence of pancreatic fistula. Therefore, there remained a need to compare the two methods following PD. Mesh-reinforced PG and mesh-reinforced PJ were compared with each other in our study. The mesh-reinforced PG showed advantages in PD in that its comprehensive complication index was lower than that of the mesh-reinforced PJ group. We also showed a significant benefit of mesh-reinforced PG reconstruction compared with mesh-reinforced PJ with respect to intra-abdominal fluid collection and delayed gastric emptying. Pancreatic and biliary fistulas also showed lower trends of incidence in mesh-reinforced PG.

There are additional factors supporting our conclusion that mesh-reinforced PG was superior to PJ. First, the blood supply to the stomach is rich, promoting healing between the stomach and the pancreatic stump [42]. Second, in the acidic gastric environment, pancreatic enzymes will not be activated, preventing deleterious tissue digestion around the anastomosis [43]. Third, a nasogastric tube can be used to decompress, and an endoscope can easily access the anastomosis when needed [43, 44]. However, the learning curve of the surgeon and the experience of the center also influence the method we chose. As a result, we suggest that mesh-reinforced PG can be performed in patients following PD to prevent postoperative complications, especially pancreatic fistulas.

There are several limitations in current study. First of all, as this study was a retrospective observational analysis, it could not exclude the impact of residual confounding factors completely, including the fact that patient distribution between groups depended on surgeon’s preference. Second, as it is just a single-center study; multicenter studies are needed for further confirmation. Third, the results lacked a control group without a mesh-reinforced technique; a randomized controlled trial would be necessary to conduct to avoid such bias in the future.

## Conclusions

The current study confirmed that the mesh-reinforced technique may be beneficial in patients following PD. Although mesh-reinforced PG did not show significant differences in pancreatic fistulas and biliary fistulas, the CCI, intra-abdominal fluid collection, and delayed gastric emptying were significantly lower in patients with the mesh-reinforced PG than in mesh-reinforced PJ. In summary, the mesh-reinforced technique is preferred during pancreaticoduodenectomy, especially mesh-reinforced pancreatogastrostomy.

## Abbreviations

BMI: Body mass index
CCI: Comprehensive complication index
PD: Pancreaticoduodenectomy
PG: Pancreatogastrostomy
PJ: Pancreatojejunostomy
POPF: Postoperative pancreatic fistula
